# Supplementary material for: A comparative work on the magnetic field-dependent properties of plate-like and spherical iron particle-based magnetorheological grease
Source: PLoS One. 2018 Apr 9;13(4):e0191795. doi: 10.1371/journal.pone.0191795 (PMC5890967; doi:10.1371/journal.pone.0191795)
Supplement: S1 File — (DOCX) [file pone.0191795.s001.docx]

*A Comparative Work on the Magnetic Field-dependent Properties of Plate-like and Spherical Iron Particle-based Magnetorheological Grease*

# Abstract

In this study, a new magnetorheological (MR) grease was made featuring plate-like carbonyl iron (CI) particles, and its magnetic field-dependent rheological properties were experimentally characterized. The plate-like CI particles were prepared through high-energy ball milling of spherical CI particles. Then, three different ratios of the CI particles in the MR grease, varying from 30 to 70 wt%, were mixed by dispersing the plate-like CI particles into the grease medium with a mechanical stirrer. The magnetic field-dependent rheological properties of the plate-like CI particle-based MR grease were then investigated using a rheometer by changing the magnetic field intensity from 0 to 0.7 T at room temperature. The measurement was undertaken at two different modes, namely, a continuous shear mode and oscillation mode. It was shown that both the apparent viscosity and storage modulus of the MR grease were heavily dependent on the magnetic field intensity as well as the CI particle fraction. In addition, the differences in the yield stress and the MR effect between the proposed MR grease featuring the plate-like CI particles and the existing MR grease with the spherical CI particles were investigated and discussed in detail.

Keywords: magnetorheological (MR) grease; particle shape; plate-like particles; spherical particles; yield stress, MR effect

# Introduction

Magnetorheological (MR) grease is classified as one of the smart materials because of its adjustable and tuneable rheological characteristics that are controlled by an external magnetic field. The rheological properties of MR grease can be changed continuously, rapidly and reversibly within milliseconds, depending on the application of the magnetic field (1, 2). At the beginning stage, MR grease was introduced to overcome the problem of sedimentation in MR fluids, which resulted in a reduction of the MR effect and the limitation of practical applications (3, 4). In other words, the grease was used as an additive that was able to eliminate the sedimentation rate of MR fluids containing a high density of iron particles. The study on this issue was continued by (5), who replaced the carrier fluid in MR fluids with grease. Specifically, they focused on the stability of the MR grease under the influence of an applied magnetic field. Thus, the magnetisable particles suspended in the grease medium formed a strong solid structure in the presence of an applied magnetic field (on-state). Mohamad et al. (6) investigated the effect of magnetisable particles of MR grease under the influence of various magnetic fields. From this study, it was found that a carbonyl iron (CI) particle fraction of 70 wt% yielded the highest yield stress (52.7 kPa) and MR effect (952.38%). The advantages of MR grease are the absence of sedimentation and its high magnetic rheological effect due to the adjustable viscosity. MR grease is basically formulated utilizing micron-sized soft magnetisable particles, with grease as the suspension medium (7). In order to improve the stability and performance of MR grease, Park et al. (2) studied the effects of a nanoparticles additive-based MR grease in various magnetic field intensities. The nanoparticles exhibited good stability without diminishing the field-dependent properties of the MR grease such as the yield stress. Instead of employing nanoparticles to improve the properties of the MR grease, Kim et al. (8) investigated the effects of kerosene oil as an additive in MR grease in off-state and on-state conditions. It was shown that the apparent viscosity and the MR effect of the MR grease decreased due to the kerosene oil. It should be noted here that the abovementioned studies on MR grease used spherical CI particles. Recently, the possibility of the shape of the particles having a significant effect on the magnetic field-dependent rheological properties of MR materials was reported in (9).

Concerns regarding the shape of the CI particles have attracted many researchers due to its significance for the improvement of stability as well as the enhancement of the magnetic field-dependent yield stress of MR materials. One of the earliest works that discussed the effects of the shape of the particles was conducted by Siebert et al. (10), where they examined the behaviour of MR fluids with plate-shaped magnetisable particles in terms of their apparent viscosity and yield stress toward the particle volume fraction. From the study, it was found that MR fluids with plate-shaped particles have a higher apparent viscosity and yield stress compared to MR fluids with spherical-shaped particles. This phenomenon is due to the expansion of the surface contact area between the plate-shaped particles caused by the reduction in total separation between the neighbouring particles. As reported in the literature (11,12), plate-like CI particles tend to reduce the interparticle gap due to their long axis and larger diameter compared to spherical CI particles. In addition, plate-like CI particles are more easily magnetized at low magnetic fields owing to their shape anisotropy. Moreover, the dipole-dipole interactions that occur in the shape anisotropy due to the stimulated magnetic field can only be attained with irregular-shaped particles. The shape anisotropy can produce one or more axes along the long axis of plate-shaped particles, depending on a favourable direction of magnetization. Therefore, a higher force is required for a fluid to flow around the particles. The study continued with an investigation by Upadhyay et al. (12) into the effects of plate-shaped particles in MR fluids under an oscillatory shear flow. The results obtained from this work showed that there was an increase in the storage modulus, which directly indicated strong solid chain structures due to the friction between the plate-shaped particles. In addition, it was shown that this behaviour was saturated at a low magnetic field intensity of 80 kA m^-1^, and that the sedimentation rate was significantly decreased compared to MR fluids consisting of spherical-shaped magnetisable particles. Shilan et al. (13) reported on the rheological properties and sedimentation of MR fluids based on plate-shaped particles and compared them with spherical particle-based MR fluids. Their results indicated that the magnetic saturation of the plate-like particles was 8% higher than that of the spherical particles. In addition, the yield stress of the plate-like particle-based MR fluids increased by up to 270%. However, the sedimentation problem still occurred in the plate-like particle-based MR fluids and the authors merely reduced the rate by up to 20% compared to the spherical particle-based MR fluids. Recently, Wei et al. (14) studied the effects of using non-spherical particles in MR grease, where computer simulations were employed to observe the rheological properties. It was shown that the MR grease using hexagon-shaped magnetisable particles had a higher magnetic field-dependent shear stress that increased from 7.4 to 21.7 kPa compared to the spherical particles (6.2 to 16.6 kPa). This simulation proved that the contact area between the particles plays a very important role in the magnetic field-dependent rheological characteristics of MR grease. It should be mentioned here that other particle geometries and properties such as size and saturation magnetization are also significant for achieving a high performances in MR grease like MR fluids (8, 15). Vicente et al. (16) claimed that non-spherical particles with the same fractions as spherical particles can have a greater magnetic intensity between the particles with the same input current. This happens due to the smaller demagnetization that occurs in the non-spherical particles. Therefore, small fractions of non-spherical particles are required to produce stronger magnetic fluids in on-state conditions. In addition, the orientational distribution and rheological properties of non-spherical particles are appropriate for use in devices that work in dynamic modes such as damping devices, vibration controls, actuators, shock absorbers, engine mounts, and in MR polishing (1, 4, 7, 17). Some of these applications require MR grease, which can produce a high yield stress and MR effect, especially in low-intensity magnetic fields (17).

As surveyed from the above literature, studies on the comprehensive effects of the shape of particles on the magnetic field-dependent rheological performance of MR grease are considerably rare. To the best of the authors’ knowledge, studies into the effects of different particle shapes on the magnetic field-dependent performance of MR grease so far have been undertaken through simulation only (14), despite many works on the plate-like iron particle-based MR fluids via both simulation and experiment (10–13, 16, 18). Consequently, the main technical contribution of this work was to experimentally investigate and compare the magnetic field-dependent rheological properties of MR grease with regard to two different particle shapes, namely plate-like CI particles and spherical CI particles. Although the plate-like particles have been previously investigated in MR fluids, the behaviour of the particles in a medium with a much higher viscosity has not been clearly investigated so far. In order to clearly observe the effect of the non-spherical CI particles in a grease medium with a higher viscosity than the carrier liquid of MR fluids, three different ratios of plate-like CI particle-based MR grease were prepared and their rheological properties were characterized in continuous and dynamic shear modes via a commercial rheometer. The rheological properties of the plate-like CI particle-based MR grease (MRG-P) were obtained and evaluated in terms of the apparent viscosity and elasticity, depending on the applied magnetic field. In addition, the dissipation energy and friction impact on the MR grease were discussed accordingly. It was also demonstrated that different rheological characteristics existed between the two different shapes of CI particles in terms of the field-dependent yield stress. As mentioned above, MR grease was initially introduced to overcome the problem of sedimentation in MR fluids. However, the issue of sedimentation between the two different shapes of CI particles was not discussed in this work.

# Preparation of MR Grease Samples

## Materials and Test Method

Spherical CI particles (OM series) purchased from BASF Germany were used to prepare the plate-like CI particles. The average size and density of the spherical CI particles were 5 µm and 7.874 g/cm^3^, respectively, as provided by the manufacturer. The plate-like CI particles were obtained from a milling process utilizing a rotary ball mill (QM-5 model from Tencan Company). The details of the procedure were undertaken according to the method described by Shilan et al. (13). In this work, the duration of the milling process was 40 hours. Zirconia balls were used as the grinding medium to modify the shape of the spherical CI particles. The preferred ball-to-powder ratio was 20:1. In order to overcome the problem of adhesion between the particles, pure ethanol was added as a process controlling agent (PCA). For the preparation of the MR grease, commercial grease (NPC Highrex HD-3 Grease, Nippon Koyu Ltd, Japan) was chosen as the suspension medium. The density and viscosity of the commercial grease, as specified by the manufacturer, were 0.92 g/cm^3^ and 190 cSt, respectively. Three different ratios of the plate-like CI particles (to be referred to as MRG-P after this), varying from 30 to 70 wt%, were prepared in a grease medium (refer to Table 1). At first, the grease was stirred for five minutes using a mechanical stirrer. Subsequently, the plate-like CI particles were added and stirred for another two hours to enhance the homogeneity of the MR grease. The process for the preparation of the samples was carried out at room temperature. The same procedure was repeated for the spherical CI particles (referred to as MRG-S after this) for comparison.

## Characterization of Rheological Properties

A field emission scanning electron microscope (JEOL, JSM-IT300) was used to observe the shape and size of the particles. The test was conducted in a high vacuum mode with a magnification of 3000x with the application of an accelerating voltage of 1 kV to the particles. The crystallinity of the CI particles was examined using an X-ray diffractometer (Empyrean, PANalytical). The X-ray patterns were evaluated using the Highscore software. In addition, a vibrating sample magnetometer was used to study the magnetic properties of the MR grease. The test was carried out using a Lakeshore 7404 series at a maximum applied magnetic field intensity of 1.2 T at 25 °C. Rotational and oscillatory shear tests were performed using a commercial parallel plate rheometer (Anton Paar, Physica, MCR302) equipped with a controlled magnetic field supported by an MR device (MRD70/1T). The magnetic field that was applied on the samples was controlled by adjusting the applied current from 0 to 5 A (refer to Table 2) instead of the magnetic field where the Tesla unit is used. By regulating the constant current value, the coil generated a constant magnetic field, where the magnetic flux density of the samples was measured using a Teslameter that was attached to the device. The direction of the magnetic field was perpendicular to the direction of the flow. The measuring plate used for the characterization had a diameter of 20 mm with a constant gap of 1 mm. For this test, 1 mL of the MR grease sample was used to fill the base plate. The measurements of the apparent viscosity and shear stress were carried out at a shear rate reaching up to 2000 s^-1^. In the meantime, the viscoelastic properties in terms of the elasticity and viscosity were evaluated under an oscillation shear mode. The oscillation shear was carried out at strain amplitude of 1% and an excitation frequency of 1.5 Hz. The temperature of the rheometer was controlled by a Viscotherm VT2 so as to maintain the temperature at 25°C throughout the experiments. After each experiment, the MR grease samples were demagnetized to keep the remanence below 1 mT before starting with a new magnetic field. In addition, the experiment was repeated more than three times to ensure the consistency of the results. The performance of the MR grease in terms of the yield stress and MR effect was then calculated.

# Experimental Results and Discussion

## Physical Properties

Figure 1(a) shows the anisometric shape of the CI particles, which can be considered to be a good approximation to a plate-like shape. After 40 hours of milling, the particles were flattened into various plate-like shapes with a more homogenous dispersion. The average size of the plate-like CI particles was 6.2 µm. On the other hand, the spherical CI particles shown in Figure 1(b) were significantly smaller (5 µm) than the plate-like particles. The effect of the different sizes and shapes of the CI particles also influenced the magnetic properties of the MR grease samples.

Figure 2 shows the XRD patterns of the spherical and plate-like CI particles at room temperature. As depicted, the XRD patterns matched the value of the CI particles, as reported in the literature (11, 12, 17, 19, 20). A series of characteristic peaks was observable at the (110), (200) and (211) planes of both shapes, and this was attributed to the reflection of the CI particles. In addition, there were no peaks that corresponded with the impurities present in the plate-like CI particles after the milling process.

A comparison of the magnetic properties of the plate-like and spherical CI particles is depicted in Figure 3. In general, the spherical CI particles had a higher magnetization compared to the plate-like particles, which were 309.75 emu/g (2.14 T) and 272.5 emu/g (2.19 T), respectively. Nevertheless, the magnetization of both shapes of CI particles had the same magnetic saturation at magnetic fields of above 0.5 T (5000 G). As depicted, the trend of this result corresponded with what has been reported in the literature, although the magnetization value obtained in this study was slightly higher (19, 21). The difference in the magnetic saturation depended on the size and shape, despite the fact that the same material was used (22). The magnetic saturation of the plate-like CI particles decreased due to the increased oxidation that occurred inside the particles during the milling process (23). In addition, the plate-like CI particles had a higher coercivity and large hysteresis loop region, as can be observed from the inset in Figure 3. Frequently, the coercivity was linearly dependent on the magnetic anisotropy and demagnetization factors, where an appropriate magnetic field was required to shift the magnetization direction, depending on the shape of the CI particles (19). Thus, the shape anisotropy of the plate-like CI particles that were magnetized more easily at a low applied magnetic field was attributed to the long configuration of their axes and the formation of stronger chain structures (24). The details of the magnetic properties of the plate-like and spherical CI particles are given in Table 3.

## Rheological Properties under Continuous Shear Mode

Figure 4 shows the measured apparent viscosity versus the magnetic flux density for the MR grease with different fractions of CI particles at a shear rate of 400 s^-1^. As expected, the apparent viscosity for both types of MR grease increased with an increase in the magnetic flux density and CI particle fraction. From the observation, the MRG-P 30 exhibited a higher apparent viscosity than the MRG-S 30. This outcome was expected to occur in the MRG-P because of the larger surface contact area, which led to the strong attraction between the plate-like CI particles (13). In addition, the strong attraction between the CI particles led to their rearrangement into lines to form chain structures that were parallel to the applied magnetic field. Besides that, the magnetization of the plate-like CI particles between 0.1 to 0.5 T was slightly higher than that of the spherical CI particles, as shown in the magnetization curves (Figure 3). The chain formations became strong and thick as the applied magnetic field increased. As a consequence, the gap between the particles decreased, resulting in the formation of hard chain structures, and indirectly increasing the apparent viscosity of the suspension (10). In addition to the inter-particle interaction, a conflict existed between the particles and the viscous force under the influence of the magnetic field. Thus, the particles moved with difficulty in the grease medium to align the chain structures under a high apparent viscosity (25). Generally, the particle volume concentration is dependent on the strength of the applied magnetic field. On the other hand, the MRG-P with 50 and 70 wt% of CI particles showed a lower apparent viscosity compared to the same fractions and magnetic flux density of the MRG-S. In this study, the particle fractions in the MRG-P influenced the saturation point due to the shape anisotropy. This phenomenon happened because the plate-like CI particles had a higher magnetization at a low magnetic field intensity (0.1 to 0.5 T), and a lower magnetic saturation when the intensity of the magnetic field was above 0.5 T compared to the spherical CI particles. In addition, the size and shape of the CI particles influenced the orientation of the particle chains under on-state conditions. By increasing the size of the CI particles, the gap between the particles was reduced, indirectly generating stronger chain structures. In conclusion, the rheological performance of the MRGP-50 and MRGP-70 declined as the magnetic field exceeded 0.5 T (3 A) as compared to the MRGP-30. Moreover, the trend of the rheological properties of the MRG**-**P was in agreement with a previous finding (12). Therefore, the shear strength of the fluid did not increase even though the strength of the magnetic field increased (22).

Furthermore, the flow curve of the shear stress for both types of MR grease with a content of 70 wt% of CI particles was examined under the influence of the shear rate, as shown in Figure 5(a). Different magnetic field strengths were applied to examine the effect on the MR grease. As can be seen in Figure 5, the graph for both types of MR grease was unstable during the off-state condition. At this state, the particles were randomly dispersed in the grease medium and easily followed the shearing direction. Meanwhile, the shear stress of the MRG-S kept fluctuating at 1 A, and gradually became stable as the applied magnetic field increased. In contrast, the shear stress pattern for the MRG-P promptly became stable in the presence of a magnetic field. Besides that, the shear stress of the MRG-P was concentrated earlier at 3 A compared to the MRG-S at 4 A with a shear rate that was less than 1000 s^-1^. This phenomenon referred to the maximum current that was applicable to the samples, which revealed that the saturation points for both shapes of CI particles started at an applied magnetic field that was higher than 0.5 T. In this study, the MRGP-70 reached its magnetic saturation at 3 A (0.628 T), while the magnetic saturation of the MRGS-70 was at 4 A (0.744 T). In addition, this could also have been related to the dipole-dipole interactions which occurred in the shape anisotropy due to the stimulated magnetic field, which can only be attained by irregular-shaped particles. The shape anisotropy can produce one or more axes along the long axis of the plate-like particles, depending on the favourable direction of magnetization. Otherwise, the demagnetization field for the shape anisotropy is more complex due to the spatial inhomogeneity of magnetization (26). As a result, a greater value of retentivity was stimulated for the plate-like particles due to their easy magnetization axis and smaller demagnetization factor in their long direction (20, 21). This occurrence was in contrast to the spherical particles, which are normally aligned based on the isotropic orientation (27).

Figure 5(b) shows the apparent viscosity patterns of the 70 wt% MRG-P and MRG-S under the influence of shear rates with the magnetic ramp up. It was found that the apparent viscosity became greater with an increase in the applied magnetic field strength, and was lowered as the shear rate increased. This behaviour demonstrated that both types of MR grease exhibited shear thinning due to the strong and fast deformation of the particle chain structures (3). Moreover, the characteristics of the grease medium also contributed to the reduction in the apparent viscosity, which could have brought about effective lubrication at a high frequency range during the motion of the lubricated surface (7, 28). As can be seen in Figure 5(b), the off-state apparent viscosity of the MRG-P was unstable compared to the off-state apparent viscosity of the MRG-S due to the very thick composition of the MR grease. It should be noted here that a slight effect occurred when the parallel plate of the rheometer slipped during shearing at a high shear rate. It is necessary to mention that the shearing condition had a greater effect on the plate-like particles compared to the spherical particles due to their larger surface contact area (11). However, the MRG-P had a higher apparent viscosity compared to the MRG-S when the magnetic field strength was 0.5 T and the applied current was 3 A. This was due to the fact that it was easy for the plate-like particles to be magnetized as they were aligned along their long axis, and it became complex to demagnetize them (19). As a consequence, faster magnetization occurred and strong solid chain structures were formed below the saturation point. It can be concluded that the stability of the MR grease was expressed under the shearing conditions.

The value of the yield stress can be determined either by extrapolating the shear stress at zero shear rate, which is known as static yield stress, or by characterizing the shear stress with respect to the rheological model, which is recognized as dynamic shear stress. In this work, the dynamic yield stress was determined using a Bingham plastic rheological model (29). In this model, the shear stress is given by

| $\tau= \tau_{y}+ \eta\dot{\gamma}$ $\tau\geq\tau_{y}$ (1) |
| --- |

where $\tau$ denotes the shear stress, $\tau_{y}$ is the yield stress as a function of the magnetic field, $\eta$ is the apparent viscosity, which represents the slope of the shear stress and shear rate curve at a higher shear rate, and $\dot{\gamma}$ is the shear rate. The Bingham model was fitted to the experimental data. The MRG-P with 30 wt% of CI particles showed the highest yield stress during the off-state and at low applied magnetic fields (< 2A) compared to the MRG-S. In the meantime, the MRG-P with 50 and 70 wt% of CI particles displayed a higher yield stress at applied magnetic fields above 3A. This was due to the high composition of the CI particles, which led to an enlargement of the surface contact area, thereby indirectly reducing the void ratio where the pre-chain structures were formed (19, 30). As the particles were fully packed with high induced magnetic polarization, the friction force became more dominant (10, 31). Therefore, a high yield stress was required to break the bonds of the chain structures so that the fluid could flow. In contrast, the yield stress of the MRG-P incorporated with 30 wt% of plate-like CI particles was reduced at a higher applied magnetic field due to the increasing void ratio, where the particles did not completely fill the grease medium, resulting in the loosening of the chains in the MR grease concentration (32). The particles were not saturated to form pre-structures even though the gap between the particles was small to allow free orientation movement, defined as the damping effect (10). Moreover, the friction force in the MRG-P became less predominant as the magnetic field strength increased due to the large surface contact area compared to the MRG-S. Thus, less yield stress was required for the fluid to flow in the MRG-P.

## Dynamic Properties under Oscillation Mode

The dynamic properties of the MRG-P and MRG-S, such as the elastic and dissipation energy, were investigated under oscillation using a shear rheometer. Figures 6(a) and (b) present the storage modulus, G’ and the loss modulus, G”, respectively, with respect to the magnetic field strength at a constant strain amplitude of γ = 1% and frequency of 1.5 Hz. The G’ and G” showed increasing trends as the magnetic flux density increased for both types of MR grease. This phenomenon indicated that the G’ seemed inclined to saturation due to the nonlinear viscoelastic behaviour at a high magnetic flux density (33). However, the G” values of the MRG-P and MRG-S were lower than the G’ values. At this point, the formation of chain structures predominated over the deformation process. It was evident that the G’ was dominantly dependent on the strength of the CI particle chains, which were formed at a higher magnetic flux density. The dipole-dipole attractive interactions between the CI particles gradually increased with an increase in the magnetic flux density, which led to the enhancement of the stiffness (10, 15, 28, 29). In comparing the two types of MR grease, the MRG-P, which was incorporated with 30 and 50 wt% of plate-like CI particles, had a higher G’ compared to the MRG-S with the same fractions. Meanwhile, the G’ of the MRG-P 70 declined compared to the MRG-S 70. This was due to the appearance of strong magnetic interactions between the particles, which indirectly reduced the void ratio at the low and medium applied magnetic fields (34). Similarly, the viscosity trend with stronger structures was less noticeable for the plate-like CI particles with high concentrations and high applied magnetic fields.

The effects of the strain amplitude on the G’ and G” for different fractions of MRG-P and MRG-S are illustrated in Figures 7(a) and (b). The applied magnetic field was kept constant at 3 A to determine the linear viscoelastic (LVE) region for both types of MR grease. The G’ of the MRG-P and MRG-S shifted to higher values with an increase in the CI particle fraction, thereby indicating strong viscoelastic behaviour (19). Based on the graph shown in Figure 7(a), it was observed that the G’ of the MRG-P dropped at strains of < 0.03%, while the G’ of the MRG-S declined at strains of < 0.1% owing to the strong Payne effect caused by the disintegration of the particle chains when a large strain amplitude was applied (35). In addition, the different shapes of the CI particles influenced the range of the strain amplitude that was applied on the samples. As can be seen in Figure 7(b), the G” was smaller for the MRG-P and MRG-S at strains of <0.1%, and suddenly increased when the strain exceeded 0.1% at the same applied magnetic field. In contrast, the G” of the MRG-S vigorously fluctuated compared to the G” of the MRG-P at a low strain. It apparently showed that the dissipation energy of the spherical-shaped CI particles was higher compared to the plate-like CI particles owing to the different shape anisotropy.

The performance of the MRG-P and MRG-S under an oscillatory shear mode was measured by the relative MR effect. In this work, the relative MR effect was calculated from the storage modulus using the following equation (36):

| $MR effect= \frac{G^{'}-{G'}_{0}}{{G'}_{0}}x100$ (2) |
| --- |

From the graph in Figure 8, the MRG-P incorporated with 30 wt% of CI particles produced an MR effect of 52.5%, which was higher than the MR effect of 46.68% of the MRG-S at a magnetic field intensity of 0.7 T. However, the MR effect of the MRG-P with high fractions of CI particles was lower compared to the MRG-S at the same applied magnetic field intensity (0.7 T). The MR effects of the MRG-P incorporated with 50 and 70 wt% of CI particles were 155.52% and 728.35%, respectively. In the meantime, the MR effects of the MRG-S with 50 and 70 wt% of CI particles were 205.94% and 952.38%, respectively. As mentioned earlier, the performance of the MR grease is dependent on the fraction, size and shape of the CI particles used as well as the applied magnetic field strength (9). The higher fractions of CI particles had a higher MR effect due to the alignment of the particles in the chain structures that became more closely packed. The chain alignment of the CI particles was more likely to be anisotropic, thereby improving the interaction of the particles due to the magnetic polarization induced between the applied magnetic field and the suspended CI particles (6, 15, 19) This phenomenon indirectly enhanced the elastic modulus, which promoted a higher MR effect.

# Conclusion

In this work, plate-like CI particles were made from spherical CI particles using the ball milling process. Different fractions of the plate-like (MRG-P) and spherical (MRG-S) CI particles were utilized for making MR grease through a mixing process to investigate their rheological properties with respect to the magnetic field. The comparative changes in the MR performance between the MRG-P and MRG-S were experimentally evaluated via a rotational and oscillatory shear rheometer. It was observed that the CI particle fraction had a significant effect on the magnetic field-dependent rheological properties, particularly under the influence of a magnetic field. The clear observations for the two different shapes of particles in the MR grease can be summarized as follows:

1) It was observed that lower volume fractions were required in the MRG-P to produce a higher yield stress and MR effect, and better lubrication properties, while higher volume fractions were required in the MRG-S. Thus, the MRG-P is more appropriate than the MRG-S for use in applications such as vibration control devices that require a high yield stress and MR effect at low magnetic fields of below 0.5 T with low fractions.

2) It was identified that the viscosity and storage modulus of the MRG-P and MRG-S increased with an increase in the magnetic field strength due to the orientation of the particles into strong chain structures. The MRG-P, in particular, had higher viscous and elastic properties at low fractions due to the magnetization of the axis, in which a reduction in the void ratio was attained. This, of course, occurred due to the larger surface contact areas of the plate-like particles. Meanwhile, the MRG-P and MRG-S had a limited LVE region at strains of above 0.03% and 0.1%, respectively. This phenomenon indirectly led to a low Payne effect.

Finally, it should be mentioned that a comparative work on the control performances of application devices using two different types of MR grease featuring plate-like and spherical CI particles will be conducted as the second phase of this work.
